# Association of Cognition-Related Genetic Polymorphisms with Elite Athlete Status: A Meta-Analysis

**DOI:** 10.3390/genes17040435

**Published:** 2026-04-09

**Authors:** Elif Akkuş, Cemre Didem Eyipınar, Yusuf Buzdagli

**Affiliations:** 1 Department of Coaching Education, Faculty of Sport Sciences, Atatürk University, Erzurum 25050, Turkey; akkuse@atauni.edu.tr; 2 Department of Physical Education and Sport, Faculty of Sport Sciences, Gaziantep University, Gaziantep 27300, Turkey; cemreeyipinar@gantep.edu.tr; 3 Department of Coaching Education, Faculty of Sport Sciences, Erzurum Technical University, Erzurum 25050, Turkey

**Keywords:** genetic polymorphism, single nucleotide polymorphism, elite athletes, sports performance, cognition-related traits

## Abstract

**Background**: Athletic performance is a multifactorial construct influenced by physiological, biomechanical, and psychological determinants. In recent years, genetic factors have been increasingly recognized as contributors to inter-individual variability in performance. In particular, polymorphisms in genes involved in neurobiological pathways have been associated with cognitive processes relevant to sport performance. However, the distribution of cognition-related genetic variants in elite athletes has not been systematically synthesized. **Methods**: This meta-analysis aimed to examine the distribution of selected candidate gene polymorphisms previously associated with cognition-related traits in elite athletes compared to control populations. A systematic literature search identified 17 eligible case–control studies investigating allele distributions of COMT rs4680, BDNF rs6265, DRD2 rs1800497, OPRM1 rs1799971, and HTR1A rs6295. Pooled analyses were performed using a fixed-effect model, and odds ratios (ORs) with 95% confidence intervals (CIs) were calculated. **Results**: Elite athletes demonstrated a significantly higher frequency of the G allele of COMT rs4680 (OR = 1.11; 95% CI: 1.02–1.21; *p* = 0.013) and the G allele of BDNF rs6265 (OR = 1.40; 95% CI: 1.10–1.77; *p* = 0.005) compared to controls. No significant differences were observed for HTR1A rs6295, DRD2 rs1800497, or OPRM1 rs1799971 polymorphisms (*p* > 0.05). **Conclusions**: This meta-analysis indicates that certain genetic variants previously associated with cognition-related traits, particularly COMT rs4680 and BDNF rs6265, are more frequently observed in elite athletes. These findings suggest a potential association between cognition-related genetic pathways and elite athletic status. However, as the present analysis is based on genetic distribution rather than direct cognitive assessments, the results should be interpreted within the context of association rather than causation.

## 1. Introduction

Athletic performance is a multifactorial construct shaped by the interaction of physiological, biomechanical, and psychological determinants. In applied settings, the identification of potential elite athletes is traditionally based on observable characteristics such as physical capacity, technical proficiency, and tactical skills, often complemented by subjective evaluations from coaches. However, accumulating evidence suggests that such approaches do not fully account for inter-individual variability in performance, highlighting the potential contribution of genetic predispositions as an underlying biological factor.

The human genome contains millions of single-nucleotide polymorphisms (SNPs), many of which have been associated with performance-related traits, including aerobic and anaerobic capacity, strength and power production, motor coordination, and muscle composition [1,2]. While early research in sports genomics primarily focused on these physiological and morphological characteristics, recent advances have extended this perspective toward neurobiological mechanisms that may influence performance through cognition-related traits. In the context of sport performance, these traits encompass a range of domain-specific processes, including decision-making speed, perceptual-cognitive expertise, executive functioning, attentional control, learning and memory processes, emotional regulation, stress responsiveness, and behavioral adaptability. These domains are particularly critical in dynamic and time-constrained sporting environments, where athletes must continuously interpret environmental cues, anticipate opponent behavior, and make rapid and accurate decisions under pressure [3,4,5].

Evidence from sports science literature indicates that elite athletes often outperform non-athletes in tasks requiring perceptual-cognitive processing and executive control. For example, perceptual-cognitive expertise enables athletes to detect and utilize relevant visual and contextual information more efficiently, thereby improving anticipation and decision-making accuracy. Similarly, executive functions such as working memory and cognitive flexibility facilitate adaptive responses to changing competitive conditions. Faster reaction times and enhanced attentional control have also been linked to superior performance in open-skill sports, including team sports and combat disciplines. These findings suggest that cognition-related traits represent a critical, yet relatively underexplored, component of elite athletic performance.

Within this framework, several candidate genes have been investigated due to their roles in key neurobiological pathways underlying these processes. In particular, catechol-O-methyltransferase (COMT), brain-derived neurotrophic factor (BDNF), dopamine receptor D2 (DRD2), μ-opioid receptor (OPRM1), and serotonin receptor 1A (HTR1A) are involved in dopaminergic, serotonergic, neurotrophic, and opioid signaling systems. These systems are known to regulate processes such as motivation, learning, emotional control, stress adaptation, and pain perception, all of which may influence performance in elite sport contexts [6,7]. The selection of these five genes in the present study was therefore based on their established roles in neurobiological pathways associated with cognition-related and behavioral traits, rather than on traditional performance-related genes linked to muscle structure or metabolism.

**Figure 1:**
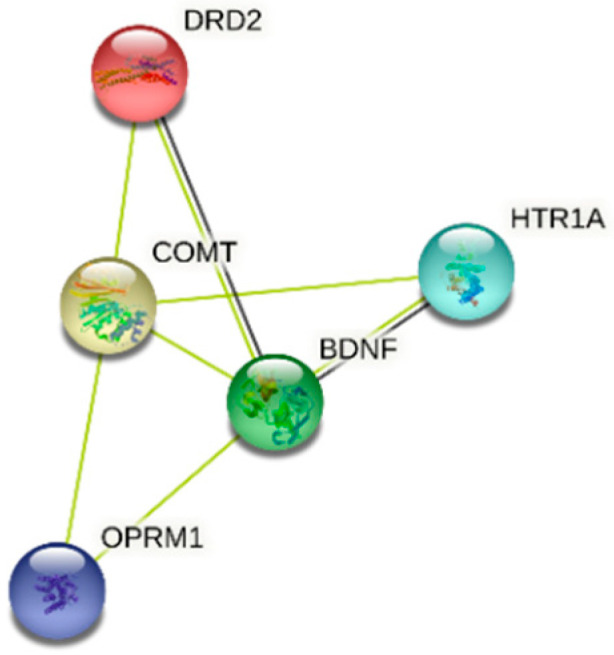
The STRING protein–protein interaction network of 5 different genes is thought to be effective in athletic performance. Colored lines between nodes (edges) show various forms of interactions, as demonstrated by gene neighborhood (green line) and co-expression in the same or distinct species (black line).

The potential interaction among these genes is illustrated in Figure 1, which presents a STRING-based protein–protein interaction network highlighting functional relationships such as gene neighborhood and co-expression patterns. This network-based representation supports the notion that these genes may not act independently but rather as part of interconnected biological systems influencing complex behavioral and cognitive processes.

The COMT gene, located on chromosome 22q11.21, encodes the catechol-O-methyltransferase enzyme (COMT; EC 2.1.1.6), which plays a central role in the degradation of catecholamines, particularly dopamine, in the prefrontal cortex. The COMT rs4680 polymorphism (Val158Met) results in a valine-to-methionine substitution that alters enzymatic activity. Individuals carrying the Met allele typically exhibit reduced COMT activity and higher synaptic dopamine availability, whereas Val allele carriers show higher enzymatic activity and lower dopamine levels. These differences have been associated with variability in executive functioning, cognitive flexibility, and information processing efficiency, although findings are not entirely consistent across populations [8,9,10,11,12].

The BDNF gene, located on chromosome 11p13, encodes a neurotrophin essential for neuronal survival, differentiation, and synaptic plasticity. The BDNF rs6265 polymorphism (Val66Met) influences activity-dependent secretion of BDNF and has been implicated in motor learning, memory formation, and synaptic adaptability. Given the role of synaptic plasticity in skill acquisition and motor learning, this polymorphism has been proposed as a potential contributor to performance-related neurocognitive processes [13,14,15,16,17,18].

The DRD2 gene, located on chromosome 11q, encodes the dopamine D2 receptor, a key component of dopaminergic neurotransmission. The DRD2 rs1800497 polymorphism has been associated with variability in receptor density and dopaminergic signaling efficiency. Previous studies have linked this polymorphism to reward-based learning and procedural learning processes, although the direction and magnitude of these associations remain inconsistent [7].

The OPRM1 gene, located on chromosome 6q25.2, encodes the μ-opioid receptor, which is involved in pain modulation, stress responses, and reward mechanisms. The OPRM1 rs1799971 polymorphism (A118G) leads to an amino acid substitution that may influence receptor function and individual sensitivity to pain and stress. These characteristics are particularly relevant in athletic contexts requiring tolerance to physical discomfort and psychological pressure [19,20].

The HTR1A gene, located on chromosome 5q11.2–q13, encodes a serotonin receptor involved in the regulation of mood, anxiety, and stress-related processes. The HTR1A rs6295 polymorphism has been associated with differences in receptor expression and emotional regulation. Given the importance of psychological stability and stress management in elite sport, this gene represents another plausible candidate in relation to cognition-related and behavioral traits [21,22].

Although these genes have been individually investigated in relation to various neurocognitive and behavioral domains, the existing literature remains fragmented and often inconsistent due to differences in study populations, sport disciplines, and methodological approaches. Moreover, most studies have been conducted at the individual level, and no previous meta-analysis has systematically synthesized the distribution of these cognition-related candidate polymorphisms in elite athletes compared to control groups. Therefore, this study aimed to systematically examine the distribution of selected candidate gene polymorphisms, COMT rs4680, BDNF rs6265, DRD2 rs1800497, OPRM1 rs1799971, and HTR1A rs6295, previously associated with cognition-related traits in elite athletes compared to controls, using a meta-analytical approach.

## 2. Methods

This study is a systematic review and meta-analysis, and it was designed according to the Preferred Reporting Items for Systematic Reviews and Meta-Analyses (PRISMA) guidelines [23].

### 2.1. Search Strategy

A systematic literature search was conducted in PubMed, EMBASE, EBSCO, and Google Scholar databases, as well as the Open Access Theses and Dissertations (OATD) database, to identify relevant studies published between January 2014 and December 2022. The search strategy was developed using a comprehensive combination of keywords and Boolean operators related to genetic polymorphisms and athletic populations. Specifically, the following search terms were applied: (“COMT” OR “BDNF” OR “DRD2” OR “OPRM1” OR “HTR1A”) AND (“polymorphism” OR “genetic variation” OR “single nucleotide polymorphism” OR “SNP”) AND (“athlete” OR “elite athlete” OR “sport performance”). The search strategy was adapted as necessary for each database to ensure optimal retrieval of relevant studies.

The time frame (2014–2022) was selected to reflect the increasing volume and methodological advancement of research in sports genomics in recent years [24].

In addition to database searches, a manual screening of reference lists from relevant articles was performed to identify additional eligible studies. All identified records were imported into a reference management system, and duplicate entries were removed before screening. All potentially eligible studies were considered regardless of access status. When full-text articles were not accessible, attempts were made to obtain the required data through institutional access or by contacting the corresponding authors. Studies for which sufficient data could not be obtained were not included in the final analysis. The study selection process was conducted in two stages. First, titles and abstracts were screened for relevance. Second, full-text articles were evaluated according to predefined inclusion and exclusion criteria. A total of 7410 records were initially identified across all sources before duplicate removal and subsequent screening.

### 2.2. Inclusion and Exclusion Criteria

Studies were considered eligible for inclusion if they met all of the following criteria: (i) employed a case–control design; (ii) investigated at least one of the following candidate polymorphisms: COMT rs4680, BDNF rs6265, DRD2 rs1800497, OPRM1 rs1799971, or HTR1A rs6295; (iii) included both an athlete group and a healthy control group; and (iv) reported sufficient genotype and/or allele frequency data to allow quantitative synthesis. Studies were excluded if they met any of the following criteria: (i) were not published in English or Turkish; (ii) did not include a control group; (iii) did not report usable genotype or allele distribution data for both athletes and controls; (iv) were review articles, meta-analyses, conference abstracts, editorials, or other non-original publications; or (v) investigated polymorphisms other than those predefined in the scope of the present meta-analysis. No exclusion was made based on publication access status in order to reduce the risk of selection bias and to ensure a more comprehensive evaluation of the available evidence.

**Figure 2:**
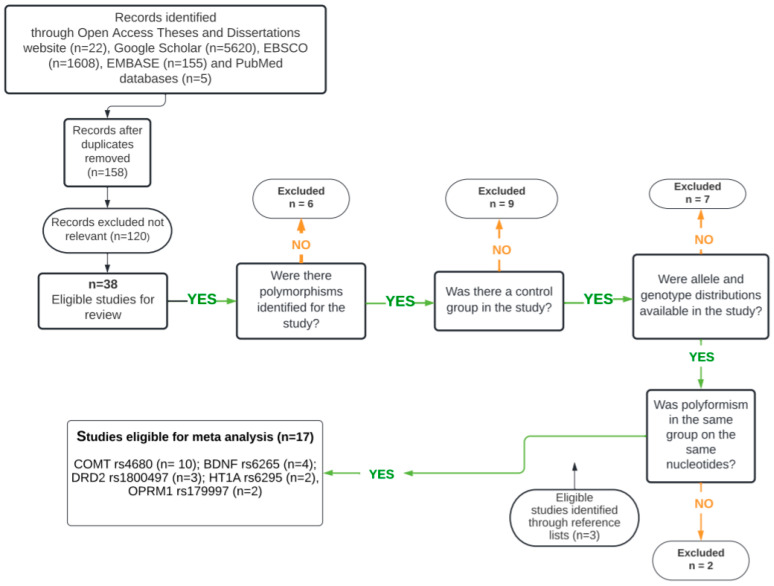
Study Selection Procedure.

A total of 7410 records were identified through database searching. After the removal of duplicate records, 158 studies remained for title and abstract screening. Of these, 120 records were excluded because they were clearly unrelated to the research question. The full texts of the remaining studies were then assessed in detail according to the predefined eligibility criteria. At this stage, 24 studies were excluded due to reasons such as irrelevant polymorphism type, absence of a control group, or insufficient genetic data for quantitative analysis. In addition, three potentially relevant studies were identified through manual screening of reference lists. Ultimately, 17 studies met the inclusion criteria and were included in the meta-analysis. The study selection process is summarized in Figure 2.

### 2.3. Data Extraction

The following information was gathered from the studies, including author and year of publication, population number, sport disciplines, gene polymorphism, genotypes, and allele distributions for both athletes and the control group. Additionally, the Hardy–Weinberg equilibrium was calculated in each study. The extracted data are presented in Table 1.

### 2.4. Statistical Analysis Process

The association between genetic polymorphisms and cognitive skills was studied using odds ratios (OR) and 95 percent confidence intervals (CI) to compare athletes to controls. Five genes and polymorphisms (COMT rs4680, BDNF rs6265, DRD2 rs1800497, OPRM1 rs179997, HTR1A rs6295) were utilized to pool odds ratios in terms of allelic models. The I^2^ test was used to assess heterogeneity [41]. MetaGenyo (https://metagenyo.genyo.es/ accessed on 15 November 2025) was used to conduct the meta-analysis. MetaGenyo is a valuable tool for performing genetic association meta-analysis [42]. Hardy–Weinberg equilibrium was calculated separately for each study. Because a divergence from the Hardy–Weinberg equilibrium might suggest a genotyping mistake or population stratification [43]. To assess the potential impact of deviations from the Hardy–Weinberg equilibrium, additional sensitivity analyses were conducted, excluding studies in which the control group showed significant deviation from HWE (*p* < 0.05). The results of these analyses were compared with the primary pooled estimates to evaluate the robustness of the findings. If the *p*-value was significant (*p* < 0.05), the genotype of the control group was considered to deviate from HWE. Egger’s regression test and funnel plot were used for investigating publication bias. In addition to the fixed-effect model, random-effects models were applied to account for potential between-study heterogeneity. The degree of heterogeneity was assessed using the I^2^ statistic, with values above 50% indicating substantial heterogeneity. Sensitivity analyses were conducted using a leave-one-out approach to evaluate the stability of the pooled effect sizes. The consistency between fixed-effect and random-effects models was examined to assess the robustness of the findings. For all analyses, *p* < 0.05 was accepted to be statistically significant.

## 3. Results

### 3.1. Characteristics of Included Studies

A total of 17 studies met the inclusion criteria and were included in this meta-analysis. It is important to note that not all studies provided data for each polymorphism investigated. Consequently, the number of studies and participants varied across SNP-specific analyses. For the COMT rs4680 polymorphism, 10 studies were included, comprising 4146 athletes and 4490 controls. The BDNF rs6265 polymorphism was analyzed based on 4 studies, including 772 athletes and 556 controls. For DRD2 rs1800497, 3 studies were available, contributing data from 596 athletes and 776 controls. The HTR1A rs6295 polymorphism was examined in 2 studies, including 266 athletes and 242 controls, while OPRM1 rs1799971 was analyzed based on 2 studies, including 826 athletes and 692 controls.

These sample sizes represent SNP-specific subsets rather than a single unified cohort. Individual studies contributed data to one or more polymorphism analyses depending on the availability of genotype and allele frequency information. Therefore, the total sample size of this meta-analysis does not correspond to a unique group of participants but reflects the cumulative data across different genetic comparisons. In addition, the included studies encompassed a heterogeneous range of athletic populations, including endurance, power, combat, and team sport athletes, as well as varying control groups (e.g., sedentary or physically active individuals). Differences in population background, sport discipline, and sample size may have contributed to between-study variability.

Regarding the Hardy–Weinberg equilibrium, a small number of studies demonstrated deviation in control groups. These studies were retained in the primary analysis to preserve statistical power but were further evaluated through sensitivity analyses to assess their potential influence on the pooled results.

### 3.2. Meta-Analysis Results

All results in this meta-analysis study were evaluated according to allele distributions. Focusing on the allelic distribution difference between the athletes and control groups, it was determined whether the relevant genes were related to cognition traits in athletes. Allelic odds ratios (OR) for five genes were calculated for this.

### 3.3. Cognition-Related Traits and the COMT Gene rs4680 Polymorphism

**Figure 3:**
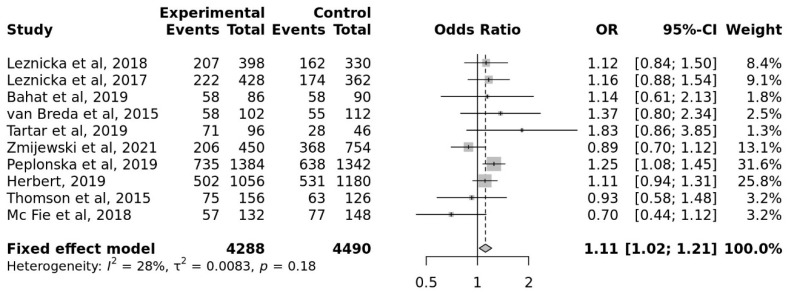
Meta-Analysis for the Association Studies for Cognition-related Traits, and COMT rs4680 (Allele G vs. A).

The G allele had a pooled OR of 1.11 compared to the A allele (95% CI, 1.02 to 1.21). The overall heterogeneity of the studies was low (I^2^ = 28%). When compared to controls, athletes showed a greater frequency of the G allele in COMT rs4680 in a statistically significant (*p* = 0.013) (Figure 3).

It can be said that the rs4680 polymorphism in the COMT gene has a strong connection with cognition-related traits because the allelic distribution of this gene in athletes is higher than in the control group.

### 3.4. Cognition-Related Traits, and BDNF Gene rs6265 Polymorphism

**Figure 4:**
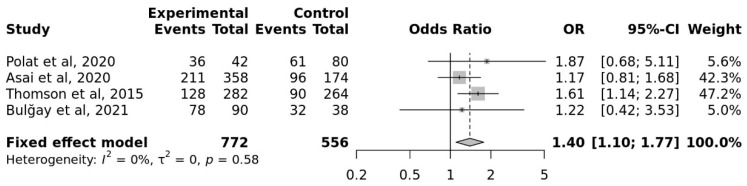
Meta-Analysis for the Association Studies for Cognition-related Traits and BDNF rs6265 (Allele G vs. A).

The G allele had a pooled OR of 1.40 compared to the A allele (95% CI, 1.10 to 1.77). The overall heterogeneity of the studies was low (I^2^ = 0%). When compared to controls, athletes showed a greater frequency of the G allele in BDNF rs6265 in a statistically significant manner (*p* = 0.005) (Figure 4). 

It can be said that the rs6265 polymorphism in the BDNF gene has a strong connection with cognitive function and cognition-related traits because the allelic distribution of this gene in athletes is higher than in the control group.

### 3.5. Cognition-Related Traits and HTR1A Gene rs6295 Polymorphism

**Figure 5:**
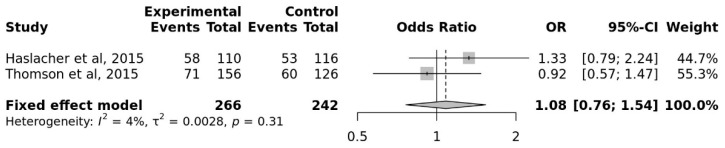
Meta-Analysis for the Association Studies for Cognition-related Traits and HTR1A rs6295 (Allele C vs. G).

The C allele had a pooled OR of 1.08 compared to the G allele (95% CI, 0.76 to 1.54). The overall heterogeneity of the studies was low (I^2^ = 4%) (Figure 5).

There was no statistically significant difference (*p* = 0.65) between the athletes and the control group regarding the distribution of the C allele associated with depression or various cognitive dysfunction. When evaluated from this point of view, the fact that the frequency of the C allele in the athletic population is not higher than in the control group is positive in terms of cognition-related traits.

### 3.6. Cognition-Related Traits and DRD2 rs1800497 and OPRM1 Gene rs179997 Polymorphisms

The G allele had a pooled OR of 2.05 compared to the A allele (95% CI, 0.83 to 5.07) for the DRD2 gene rs1800497 polymorphism. The A allele had a pooled OR of 1.01 compared to the G allele (95% CI, 0.7 to 1.4) for the OPRM1 gene rs179997 polymorphism. While the overall heterogeneity of the studies was low (I^2^ = 0%) for the OPRM1 gene rs179997 polymorphism, for the DRD2 gene rs1800497 polymorphism, the heterogeneity level was (I^2^ = 76%). Additionally, all statistics of the five genes associated with cognition-related traits are summarized in Table 2.

In brief, the distribution of alleles associated with cognition-related traits did not differ (for DRD2 rs1800497, *p* = 0.11 and OPRM1 rs179997, *p* = 0.92) between athletes and control groups, and it can be said that DRD2 rs1800497 and OPRM1 rs179997 gene polymorphisms are not associated with cognition-related traits in athletes.

To further assess the robustness of the findings, random-effects analyses were performed for all polymorphisms. The results obtained using the random-effects model were largely consistent with those of the fixed-effect model, particularly for COMT rs4680 and BDNF rs6265 polymorphisms, where significant associations remained unchanged.

However, for the DRD2 rs1800497 polymorphism, which demonstrated substantial heterogeneity (I^2^ = 76%), the random-effects model yielded wider confidence intervals and reduced statistical precision, further supporting the need for cautious interpretation.

Sensitivity analyses using a leave-one-out approach indicated that no single study disproportionately influenced the overall effect estimates. To further evaluate the influence of Hardy–Weinberg equilibrium deviations, sensitivity analyses were performed by excluding studies in which the control groups deviated from HWE. The exclusion of these studies did not materially alter the overall effect estimates for COMT rs4680 and BDNF rs6265 polymorphisms, and the direction and significance of the associations remained consistent.

These findings suggest that the main results are robust and not substantially influenced by potential biases related to HWE deviation.

### 3.7. Publication Bias

According to Table 2, Egger’s test *p* values (as *p* > 0.05) indicate that there is no evidence of publication bias among studies [44]. Because there was insufficient research using the HTR1A and OPRM1 genes, Egger’s regression test value for these studies could not be determined. Figure 5 shows funnel plots of all studies with three genes, COMT, BDNF, and DRD2, respectively.

According to Table 2, Egger’s test did not indicate significant asymmetry for COMT, BDNF, and DRD2 polymorphisms (*p* > 0.05). However, it is important to note that the number of included studies for several polymorphisms was limited. Therefore, the statistical power of Egger’s test and the interpretability of funnel plots are restricted. For HTR1A and OPRM1 polymorphisms, publication bias could not be reliably assessed due to the small number of available studies. Accordingly, the results of publication bias analyses should be interpreted with caution.

**Figure 6:**
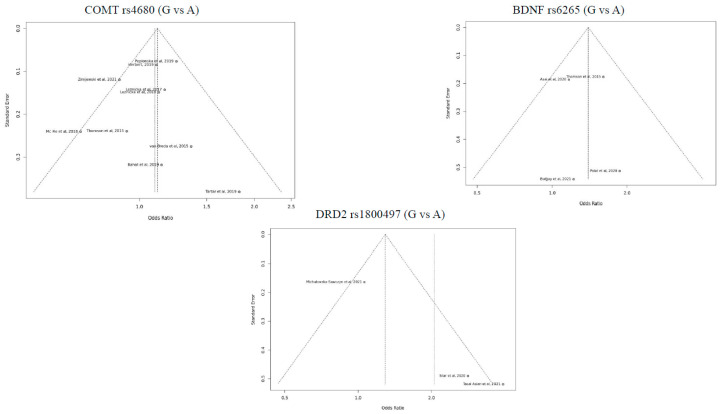
Funnel Plot of Included Studies.

Since the funnel plot of the three genes is symmetrical, it can be said that there is no publication bias (Figure 6).

## 4. Discussion

To our knowledge, this is the first meta-analysis to examine the distribution of selected candidate polymorphisms previously associated with cognition-related traits in athlete populations. The present findings indicate that the G allele of COMT rs4680 and the G allele of BDNF rs6265 were more frequently observed in elite athletes than in control groups, whereas no significant between-group differences were found for DRD2 rs1800497, OPRM1 rs1799971, or HTR1A rs6295. These findings should be interpreted within the framework of genetic association rather than direct functional outcomes, as the present study compared allele distributions between athlete and control groups and did not include direct neuropsychological or behavioral assessments.

It is also important to clarify that the term cognition-related traits in the present study does not refer to a single unified construct. Rather, it encompasses a range of interrelated domains that have been linked in the literature to the investigated genes, including executive functioning, learning and memory processes, emotional regulation, stress responsiveness, pain perception, and related behavioral tendencies. Therefore, the present results should not be interpreted as evidence of a direct association with “cognitive performance” per se, but rather as indicating that certain genetic variants previously linked to these broader neurocognitive and behavioral domains may be more prevalent in elite athletes.

With respect to COMT rs4680, previous human studies have suggested that this polymorphism may contribute to inter-individual differences in executive functioning, cognitive flexibility, working memory, and prefrontal information processing [8,45,46]. In particular, the Met allele has been associated with lower COMT enzymatic activity and, consequently, higher dopamine availability in the prefrontal cortex, which may influence executive processing efficiency [9,10,11,12]. Studies such as that of de Frias et al. reported that Met/Met carriers performed better on selected executive and visuospatial tasks than Val allele carriers [46], while other studies also linked the Met allele to stronger performance in working memory and related cognitive domains [47,48]. In this context, the higher frequency of the G allele observed in athletes in the present meta-analysis may reflect a population-level enrichment of a variant previously examined in relation to dopaminergic regulation and cognition-related traits. However, because the present meta-analysis did not evaluate executive performance directly, these findings should not be interpreted as evidence that COMT rs4680 confers a direct cognitive or athletic advantage.

A similar interpretative caution applies to BDNF rs6265. BDNF plays a central role in neuronal differentiation, synaptic plasticity, and learning-related neurobiological processes [13,14,15,16,17,18,49,50,51,52,53]. Previous studies have associated this polymorphism with variability in memory, executive functioning, and motor learning, although the direction and magnitude of these associations have not been fully consistent across populations [49,50]. In our meta-analysis, the G allele of BDNF rs6265 was significantly more frequent in athletes than in controls. This finding may be consistent with the possibility that neurotrophic pathways related to synaptic plasticity and adaptive neural responses are relevant in elite athletic populations. Nevertheless, the current data do not allow direct conclusions regarding specific phenotypes such as memory capacity, attentional control, or motor learning efficiency in athletes. Accordingly, the present result should be interpreted as an association between athlete status and a candidate variant previously linked to cognition-related traits, rather than as evidence of a direct functional effect.

For DRD2 rs1800497, no statistically significant association was observed between athletes and controls in the pooled analysis. This is noteworthy because previous literature has linked DRD2-related dopaminergic signaling to reward processing, procedural learning, and certain affective or behavioral outcomes [7,54,55,56]. Some studies have suggested that the A allele may be associated with lower receptor availability and altered dopaminergic responsiveness, which in turn may influence neurobehavioral characteristics. However, in the present meta-analysis, the absence of a significant between-group difference indicates that the available evidence does not support a robust association between DRD2 rs1800497 and elite athlete status. This interpretation should also remain cautious because heterogeneity for this polymorphism was higher than for the other analyses, suggesting that differences in study populations, sport disciplines, and methodological characteristics may have influenced the pooled estimate. Therefore, rather than concluding that DRD2 is unrelated to performance-relevant neurobiological traits, it may be more accurate to state that current evidence is insufficient to demonstrate a consistent association in athlete populations.

The findings for OPRM1 rs1799971 should be interpreted in a similarly cautious manner. The μ-opioid receptor has been implicated in pain modulation, stress responsivity, and reward-related processes, all of which may be relevant in sporting contexts characterized by physical discomfort, pressure, and repeated exposure to demanding stimuli [19,20]. Previous reports have suggested that OPRM1 variation may be related to differences in pain sensitivity and coping style under stressful conditions [20,57,58,59]. However, our pooled analysis did not reveal a significant difference between athletes and controls for this polymorphism. Thus, while OPRM1 remains biologically plausible as a candidate gene in relation to stress- and pain-related behavioral traits, the present findings do not provide evidence for a consistent association with athlete status. Any stronger interpretation would exceed the scope of the available data.

Likewise, no significant difference was identified for HTR1A rs6295 between athletes and controls. The HTR1A receptor has been associated in the literature with mood regulation, anxiety-related traits, and stress responsiveness [60,61,62,63,64]. Because these domains are relevant to competitive environments, HTR1A has been considered a plausible candidate gene in sports-related behavioral research. However, the current meta-analysis does not support a statistically significant association between HTR1A rs6295 and athlete status. This null finding should be considered in light of the limited number of available studies and the relatively narrow evidence base. Therefore, the lack of significance in the pooled analysis should not be interpreted as definitive evidence of no relationship, but rather as indicating that current evidence remains insufficient and requires further investigation in larger and more diverse athlete cohorts.

Taken together, the present findings suggest that certain polymorphisms previously examined in relation to dopaminergic and neurotrophic mechanisms, particularly COMT rs4680 and BDNF rs6265, may be more frequent in elite athletes than in controls. At the same time, the absence of significant associations for DRD2 rs1800497, OPRM1 rs1799971, and HTR1A rs6295 highlights the importance of avoiding overly broad or deterministic interpretations in sports genomics research. Athletic performance is a highly complex phenotype shaped by multiple biological, environmental, psychological, and training-related factors. It is therefore unlikely that any single polymorphism can meaningfully explain elite status on its own.

From a methodological perspective, the interpretation of the present results should also consider several limitations. First, the number of available studies was limited for some polymorphisms, particularly HTR1A and OPRM1, which restricts statistical power and limits the strength of inference. Second, heterogeneity across studies in terms of sport discipline, ethnicity, sample size, and control-group characteristics may have influenced pooled estimates. Third, because this meta-analysis was based on genotype and allele frequencies rather than direct phenotypic assessments, it cannot determine whether the investigated variants are associated with specific neurocognitive or behavioral outcomes in athletes. These points reinforce the need for cautious interpretation and align with the view that the current findings reflect population-level genetic associations, not direct mechanistic or performance effects.

Future studies should aim to integrate genetic profiling with direct assessments of neurocognitive and behavioral phenotypes, such as executive function, decision-making, attentional control, stress regulation, and pain tolerance, in well-characterized athlete populations. Such an approach would provide a more precise understanding of whether and how these candidate polymorphisms are functionally relevant in sport settings. In addition, larger multi-center studies and meta-analyses including more homogeneous sport subgroups may help clarify whether the observed associations differ according to discipline-specific demands.

In conclusion, the present meta-analysis supports a possible association of COMT rs4680 and BDNF rs6265 with elite athlete status, while no consistent associations were observed for DRD2 rs1800497, OPRM1 rs1799971, or HTR1A rs6295. These findings contribute to the emerging literature on sport genomics by highlighting potentially relevant cognition-related candidate polymorphisms, but they should be interpreted cautiously and without implying direct cognitive or functional superiority.

## 5. Conclusions

This meta-analysis provides a comprehensive synthesis of current evidence regarding the distribution of selected candidate polymorphisms associated with cognition-related traits in elite athletes. The findings indicate that the G alleles of COMT rs4680 and BDNF rs6265 are more frequently observed in athlete populations than in controls, whereas no consistent differences were observed for HTR1A rs6295, DRD2 rs1800497, and OPRM1 rs1799971.

These results suggest that genetic variants involved in dopaminergic and neurotrophic pathways may be differentially represented in elite athletes, potentially reflecting underlying biological mechanisms related to adaptation, learning processes, and behavioral regulation in high-performance environments. However, given that the present study is based on allele-frequency comparisons rather than direct phenotypic measurements, the findings should be interpreted strictly in the context of genetic association.

Importantly, the absence of significant findings for certain polymorphisms underscores the multifactorial and complex nature of athletic performance, which cannot be attributed to a single genetic variant. Instead, performance outcomes likely emerge from the interaction of multiple genetic, environmental, and training-related factors.

Future research should move beyond association-based approaches by integrating genetic data with direct assessments of neurocognitive, psychological, and performance-related phenotypes. Such integrative designs may provide a more precise understanding of the functional significance of these polymorphisms and their potential role within the broader framework of athlete development.

## 6. Limitations

Several limitations should be considered when interpreting the findings of this meta-analysis.

➢ In addition, although efforts were made to implement a comprehensive search strategy, certain limitations related to the literature search should be acknowledged. The applied time restriction (2014–2022) may have limited the inclusion of earlier relevant studies. Furthermore, although all potentially eligible studies were considered regardless of access status, some studies could not be included due to the inability to obtain full-text articles or sufficient data, despite attempts to access them through institutional resources or by contacting the corresponding authors.
➢ The number of included studies was limited for certain polymorphisms, particularly HTR1A rs6295 and OPRM1 rs1799971. This restricted the statistical power of the analyses and limited the robustness of the pooled estimates. In addition, the small number of studies for these polymorphisms prevented a reliable assessment of publication bias and may have reduced the precision of the conclusions.
➢ Heterogeneity across the included studies should be taken into account. The analyzed studies differed in terms of sport disciplines, population characteristics, ethnic backgrounds, and sample sizes. Such variability may have influenced the pooled results and complicated direct comparisons across studies.
➢ Some included studies showed deviation from the Hardy–Weinberg equilibrium in control groups, which may reflect potential issues such as population stratification or genotyping errors. Although sensitivity analyses indicated that the overall findings were not substantially affected, this factor should still be considered when interpreting the results.
➢ The present meta-analysis was based on genotype and allele frequency data rather than direct measurements of neurocognitive or behavioral outcomes. Therefore, the findings reflect associations at the genetic level and do not allow for direct inferences regarding cognition-related traits or athletic performance.
➢ Although multiple statistical approaches, including random-effects models and sensitivity analyses, were applied to improve robustness, the overall conclusions remain dependent on the quality and availability of the included studies.

Future research should focus on larger, well-characterized cohorts and integrate genetic data with direct neurocognitive and behavioral assessments in order to clarify the functional relevance of these polymorphisms in athletic populations.

## 7. Protocol Registration

The protocol for this meta-analysis was prospectively registered in the Open Science Framework (OSF) to ensure transparency and reproducibility of the research process. The registration is available at https://osf.io/3s9gf (accessed on 15 April 2022) (DOI: 10.17605/OSF.IO/48XDM), with the date of registration recorded as 15 April 2022. The registered protocol includes details of the research objectives, eligibility criteria, and planned analytical approach.

## Figures and Tables

**Table 1:** Distribution of Genotype and Allele Frequencies of Performance-Related Gene Polymorphisms in Athletes and Control Populations Across Different Sport Disciplines.

Author, Year	Samples	*n*	Gene and Polymorphism	Genotypes	Genotypes	Genotypes	Allele	Allele	Hardy–Weinberg Value (*p*)
Zmijewski et al., 2021 [25]	Elite Swimmers Sedentary Controls	225 377	COMT rs4680	AA A:71 C:106	AG A:102 C:174	GG A:52 C:97	A A:244 C:386	G A:206 C:368	0.138
Tartar et al., 2020 [26]	Combat Athletes Athletes Non-Athlete Controls	25 23 23	COMT rs4680	AA A:0 A2:0 C:0	AG A:12 A2:13 C:18	GG A:13 A2:10 C:5	A A:12 A2:13 C:18	G A:38 A2:33 C:28	**0.002**
van Breda et al., 2015 [27]	Ironman Triathletes Active Controls	51 56	COMT rs4680	AA A:14 C:20	AG A:16 C:17	GG A:21 C:19	A A:44 C:57	G A:58 C:55	**0.0033**
Bahat et al., 2019 [28]	Ski Runners Swimmers Sedentary Controls	19 24 45	COMT rs4680	AA A:0 A2:0 C:4	AG A:15 A2:13 C:24	GG A:4 A2:11 C:17	A A:15 A2:13 C:32	G A:23 A2:35 C:58	0.2719
Mc Fie et al., 2018 [29]	Elite Rugby Athletes Controls	66 74	COMT rs4680	AA A:26 C:22	AG A:23 C:27	GG A:17 C:25	A A:75 C:71	G A:57 C:77	0.206
Leźnicka et al., 2018 [20]	Combat Athletes Healthy Men	199 165	COMT rs4680 OPRM1 rs1799971	AA A:45 C:41 AA A:167 C:136	AG A:101 C:86 AG A:30 C:29	GG A:53 C:38 GG A:2 C:0	A A:191 C:168 A A:364 C:301	G A:207 C:162 G A:34 C:29	0.5827 0.2159
Leźnicka et al., 2017 [30]	Combat Athletes Non-Athlete Controls	214 181	COMT rs4680 OPRM1 rs17999971	AA A:50 C:47 AA A:178 C:149	AG A:106 C:94 AG A:34 C:32	GG A:58 C:40 GG A:2 C:0	A A:206 C:188 A A:390 C:330	G A:222 C:174 G A:38 C:32	0.5883 0.2159
Peplonska et al., 2019 [31]	Elite Endurance Athletes Sedentary Controls	621 671	COMT rs4680	AA A:172 C:187	AG A:306 C:330	GG A:143 C:154	A A:650 C:704	G A:660 C:638	0.7167
Thomson et al., 2015 [32]	High-Risk Sportsmen Controls	141 132	COMT rs4680 BDNF rs6265 HTR1A rs6295	AA A:22 C:18 AA A:2 C:3 CC A:17 C:24	AG A:37 C:27 AG A:24 C:30 GC A:37 C:32	GG A:19 C:18 GG A:52 C:30 GG A:24 C:17	A A:81 C:63 A A:28 C:36 C A:71 C:80	G A:75 C:63 G A:128 C:90 G A:85 C:66	0.2568 **0.00** 0.8853
Herbert, 2019 [33]	Marathon Runners Non-Athlete Controls	528 559	COMT rs4680	AA A:137 C:162	AG A:280 C:263	GG A:111 C:134	A A:554 C:587	G A:502 C:531	0.0157
Haslacher et al., 2015 [34]	Endurance Athletes Controls	55 58	HTR1A rs6295	CC A:15 C:10	GC A:28 C:33	GG A:12 C:15	C A:58 C:53	G A:52 C:83	0.5294
Polat et al., 2020 [35]	Volleyball Players Controls	21 40	BDNF rs6265	AA A:0 C:2	AG A:6 C:15	GG A:15 C:23	A A:6 C:19	G A:36 C:61	0.823
Asai et al., 2020 [36]	Swimmers Judo Athletes Non-athlete Controls	105 74 87	BDNF rs6265	AA A:18 A2:6 C:19	AG A:65 A2:34 C:40	GG A:22 A2:34 C:28	A A:101 A2:46 C:78	G A:109 A2:102 C:96	0.5107
Bulğay, 2021 [37]	Jumping Athletes Middle Distance Athletes Long Distance Athletes Controls	17 15 13 19	BDNF rs6265	AA A:0 A2:0 A3:0 C:0	AG A:7 A2:2 A3:3 C:6	GG A:10 A2:13 A3:10 C:13	A A:7 A2:2 A3:3 C:6	G A:24 A2:28 A3:23 C:32	0.4138
Michałowska-Sawczyn et al., 2021 [38]	Combat Athletes Healthy Controls	258 284	DRD2 rs1800497	AA A:4 C:7	AG A:75 C:82	GG A:179 C:195	A A:83 C:96	G A:433 C:472	0.6383
Tacal Aslan et al., 2021 [39]	Football Players Controls	21 52	DRD2 rs1800497	AA A:0 C:9	AG A:5 C:18	GG A:16 C:25	A A:5 C:36	G A:37 C:68	0.1346
Silar et al., 2020 [40]	Cyclists Sedentary controls	19 52	DRD2 rs1800497	AA A:0 C:9	AG A:6 C:18	GG A:13 C:25	A A:6 C:36	G A:32 C:68	0.1346

Abbreviations: A: Athlete, A2 and A3: 2nd and 3rd place athletes, BDNF: Brain-Derived Neurotrophic Factor, C: Control, COMT: Catechol-O-Methyltransferase; DRD2: Dopamine Receptor D2; HTR1A: Hydroxytryptamine Receptor 1A, OPRM1: μ-Opioid Receptor. Bold texts indicate statistical significance level (*p* < 0.05).

**Table 2:** Statistic Summary for All Studies.

		Association Test			Models	Heterogeneity		Publication Bias
Allelic Model	Number of Studies	OR	95% CI	*p*-Value		I^2^	*p*-Value	Egger’s Test *p*-Value
COMT rs4680 (G vs. A)	10	1.11	[1.02–1.21]	0.01	Fixed	0.28	0.18	0.72
BDNF rs6265 (G vs. A)	4	1.40	[1.1–1.7]	0.005	Fixed	0	0.58	0.87
HTR1A rs6295 (C vs. G)	2	1.08	[0.7–1.5]	0.65	Fixed	0.04	0.3	NaN
DRD2 rs1800497 (G vs. A)	3	2.05	[0.83–5.07]	0.11	Fixed	0.76	0.01	0.07
OPRM1 rs179997 (A vs. G)	2	1.01	[0.7–1.4]	0.94	Fixed	0	0.92	NaN

## Data Availability

All data analyzed in this study are included in the published articles cited in the reference list. The datasets generated and/or analyzed during the current study are available from the corresponding author upon reasonable request.
